# The Battle of the Clubs

**Published:** 1896-07-11

**Authors:** 


### The Battle of the Clubs.

The subject of co-operative and provident modes of payment for medical attendance, which has now for more than a year occupied so large a place in medical politics, is a matter of far more than mere professional interest. To hundreds of hard-working professional men the development of the club system in the form which it has recently assumed has not only meant a diminution of income, small enough to begin with, but has greatly increased the amount of work which has had to be done to earn that smaller living. But it is impossible to shut one's eyes to the fact that the continued grind of extensive club practice also tends directly to the degradation of scientific medicine and the encouragement of methods of routine.

The friendly societies and the members of the various clubs who are endeavouring to drive such hard bargains with their doctors should, then, bear in mind that, after all, they will get only what they pay for, and that the ultimate effect of their operations will be to train up for their own use a class of practitioner who must from the very exigencies of the case become every year less and less reliable. It is a strange outcome of modern progress and a curious satire on the claims of the so-called working classes, that while men of light and leading are doing their utmost to obtain even for the poorest the best of medical help in sickness, the cream of the working classes, the thrifty and the self-reliant, should hold to the doctiine that a halfpenny doctor is good enough for them.

But it must always be understood that in accepting from working people such small sums, something between half a crown and four shillings a head per year, for medical attendance, the doctor only does so lest worse things befall him in the way of bad debts, and in no way because he thinks he will make his living thereby. This fact, that the ordinary club fees do not in themselves pay and are in reality only a means of protecting the doctor from loss in respect to his poorer patients, is at the bottom of the whole question, and is the real reason why medical men object as they do to the admission to these clubs of people who are in better circumstances. The doctor lives on his private practice, and so long as he can do so the poor get the benefit of club attendance at a nominal rate; but once knock away private practice, as the friendly societies and medical aid associations tend to do, and clubs, friendly societies, and all go down together, and the medical science at the service of the people is reduced to just what their halfpenny a week will pay for—which is not much.

That the provident system of payment for medical attendance is an absolute necessity for certain classes we can have no doubt, nor need we hesitate to express the opinion that the same system might possibly be extended to much higher classes of society than at present commonly avail themselves of it. But the payment must in such case be proportionate to the services demanded, and must be calculated on such a basis as shall enable medical men to live on it, to obtain from it a proper return for the time and the money which have been expended on their training and education, and to secure such leisure for study as shall prevent their drifting into routine.

In any action which the medical profession may think fit to take in regard to this question it will be well to keep clearly in view that the provident system of payment is essential to certain classes of the community both for their own sakes and as the only method by which the medical profession can hope to obtain from them any payment at all. From these the lowest fees may properly be accepted which will protect from loss, on condition that such clubs or societies are kept rigidly to the classes for whose use they are intended. In regard to all other classes, however, the principle should be definitely adhered to that the payment should be such as would afford a proper livelihood, even if the doctor had to live by that sort of work alone; and in all schemes alike the management of the clubs, societies, or associations should be kept as far as possible under the control of the profession; otherwise that will happen which in too many cases has already happened—the doctor will become the mere servant of the association under which he works, and all that good repute which has hitherto been the personal stock in trade of the family practitioner, on which in many cases he has lived comfortably far into a respected old age, will become the mere asset of a trading company which will turn him adrift so soon as his joints get stiff.

